# Dose-Dependent Toxic Effect of Cotinine-Verified Tobacco Smoking on Systemic Inflammation in Apparently Healthy Men and Women: A Nationwide Population-Based Study

**DOI:** 10.3390/ijerph16030503

**Published:** 2019-02-11

**Authors:** Won-Jun Choi, Ji-Won Lee, A Ra Cho, Yong-Jae Lee

**Affiliations:** 1 Department of Family Medicine, Yonsei University College of Medicine, 50-1 Yonsei-ro, Seodaemoon-gu, Seoul 06273, Korea; slashwj@gmail.com (W.-J.C.); indi5645@yuhs.ac (J.-W.L.); ara1713@yuhs.ac (A.R.C.); 2 Department of Medicine, Graduate School, Yonsei University, 50-1 Yonsei-ro, Seodaemoon-gu, Seoul 03722, Korea

**Keywords:** tobacco smoking, cotinine, leukocyte count, inflammation

## Abstract

Chronic exposure to tobacco smoke leads to chronic low-grade systemic inflammation; however, little is known about the dose-dependent toxic effect of objective tobacco smoking on systemic inflammation. We examined the dose-dependent toxic effect of cotinine-verified tobacco smoking on leukocyte count in a representative sample of Korean adults, including 8655 men and 10,432 women aged 19–65 years from the Korean National Health and Nutrition Examination Survey. The participants were categorized into five groups by the level of urine cotinine (Ucot) as follows: A non-smoker group (<50.00 ng/mL) and four quartile groups for smokers for both men and women. The odds ratios (ORs) and 95% confidence intervals (CIs) for sex-specific high leukocyte count (≥75th percentile) were calculated across five groups of Ucot concentrations using multiple logistic regression analyses. The mean values of leukocyte count gradually increased according to the grade of Ucot concentration in both sexes. The ORs (95% CIs) for high leukocyte count in the 4th Ucot quartile concentrations versus the non-smokers were 4.09 (3.39–4.94) for men and 3.91 (2.82–5.41) for women after adjusting for confounding variables. The present study confirmed the dose-response toxic effect of tobacco smoking on chronic low-grade systemic inflammation.

## 1. Introduction

Tobacco smoking is a major risk factor for morbidity and mortality from cardiovascular disease (CVD) [1], which is now increasingly viewed as inflammatory disease [2]. Leukocyte count is a simple, easy to obtain, and economical marker of systemic inflammation for clinical practice. A higher leukocyte count, even within the normal range, has been associated with increased incidence risk of hypertension, type 2 diabetes mellitus, CVD, all-cause mortality risk, and cancers [2,3,4,5].

Many toxins in tobacco such as nicotine, carbon monoxide, benzene, and many other reactive oxidants provoke systemic inflammation by a variety of mechanisms [6]. For the acute phase of smoking, activation of airway epithelial cells stimulates immune cells such as neutrophils and macrophages in the respiratory system, whereas chronic exposure to tobacco smoke leads to chronic low-grade systemic inflammation [7,8].

Although previous studies have reported that several inflammatory markers are increased in current smokers, most studies used a self-reported questionnaire such as smoking status (never, ex-smoker, and current smokers) or the average number of cigarettes smoked per day [9,10,11]. However, self-reported tobacco smoking may not always represent an individual’s true smoking status, because the smoking rate might be underestimated particularly in females due to the possibility of false response and recall bias [12,13].

Cotinine, a specific metabolite of nicotine in tobacco, is widely used as a quantitative biomarker of tobacco smoke exposure [14]. Due to its long half-life of approximately 16–20 h in the body [15], cotinine is detectable for several days after tobacco smoking. For this reason, the dose-dependent risk estimates for detrimental clinical outcomes related to tobacco smoking have been evaluated using cotinine (Ucot) concentrations [16,17,18]. To date, little is known about the dose-dependent toxic effect of objective tobacco smoking on systemic inflammation. Thus, we examined the dose-dependent toxic effect of cotinine-verified tobacco smoking on leukocyte count, a useful systemic inflammatory marker in clinical practice, from a representative sample of Korean adult men and women.

## 2. Materials and Methods

### 2.1. Study Population

This study was based on data obtained from the 2008–2015 Korean National Health and Nutrition Examination Survey (KNHANES), a cross-sectional and nationally representative survey conducted by the Korean Ministry of Health and Welfare. The target population of KNHANES represents non-institutionalized civilians who are aged older than 1 year in Korea. Citizens were informed that they had been randomly selected as a household to voluntarily participate in a nationally representative survey conducted by the Korean Ministry of Health and Welfare (KCDC) and that they had the right to refuse to participate in accordance with the National Health Enhancement Act supported by the National Statistics Law of Korea. The participants provided written informed consent to participate in the study. The Korea Centers for Disease Control and Prevention obtained consent from participants to use blood samples for additional academic purposes. The KNHANES was approved by the Institutional Review Board of the KCDC (IRB No. 2008-04EXP-01-C, 2009-01CON-03-2C, 2010-02CON-21-C, 2011-02CON-06-C, 2012-01EXP-01-2C, 2013-07CON-03-4C, and 2013-12EXP-03-5C). We extracted 27,734 participants (13,034 men and 14,700 women) aged 19–65 years whose Ucot data were available from the 2008, 2009, 2010, 2011, 2014, and 2015 KNHANES datasets. We excluded participants who met at least one of the following criteria (n = 8647): A history of cancer, coronary artery disease, cerebrovascular disease, pulmonary tuberculosis, asthma, and chronic obstructive lung disease, rheumatologic disease, liver cirrhosis, or chronic kidney disease; electronic cigarette users; subjects with nicotine replacement therapy; non-smokers with second-hand tobacco exposure at the workplace and home; subjects with leukocyte counts ≥10,000 cells/μL; and those with missing data or those who did not fast for 12 h prior to testing. Following these exclusions, 19,087 participants (8655 men and 10,432 women) were included in our final analysis.

### 2.2. Data Collection

The sampling units were households selected through a stratified, multistage, probability-based sampling design based on geographic area, sex, and age group using household registries. Sampling weights indicating the probability of being sampled were assigned to each participant, thus producing results that represent the entire Korean population. Participants completed four parts of a questionnaire that consisted of a health interview survey, a health behavior survey, a health examination survey, and a nutrition survey. Trained staff reviewed the completed questionnaires and entered responses into a database.

Self-reported cigarette smoking, alcohol consumption, and physical activity were determined from the questionnaires. Each participant was categorized as a non-smoker, ex-smoker, or current smoker with respect to smoking status. Participants were also asked about the frequency of alcohol intake and leisure-time physical activity on a weekly basis. Alcohol intake was divided into none, ≤once per week, and ≥two times per week. Regular exercise was divided into three groups according to frequency of moderate intensity exercise: None, ≤two times per week, and ≥three times per week. Educational level was classified as middle school or below, high school, and college or above. Household income was classified into four quartiles from the lowest to the highest. Occupation was classified into three categories: Unemployed (retired, students, and housewives); blue-collar workers (agriculture, forestry, fishery workers, craft and related trade workers, plant and machine operators and assemblers, and elementary occupations); white-collar workers (managers, professionals, technicians, clerks, and service/sales workers) according to the Korean Standard Classification of Occupations.

The health examinations included a medical history, a physical examination, and anthropometric and biochemical measurements. If participants were being treated for any disease, they were asked for data regarding their diagnosis as well as a list of medications currently being taken. Physical examinations were performed by trained medical staff following standardized procedures. Height and weight were recorded to the nearest 0.1 cm (Seca 225, Seca, Hamburg, Germany) and 0.1 kg, respectively (GL-6000-20, G-tech, Seoul, Korea). Body mass index (BMI) was calculated as the ratio of weight (kg)/height (m)^2^. Systolic and diastolic blood pressure were measured using the patient’s right arm with a standard mercury sphygmomanometer (Baumanometer; W. A. Baum Co Inc., Copiague, NY, USA). Mean arterial pressure was calculated using the equation (SBP + 2*DBP)/3. Leukocyte count was determined by an automated blood cell counter (XE-2100D, Sysmex, Kobe, Japan). Fasting plasma glucose, triglyceride, and high-density lipoprotein (HDL) cholesterol levels were measured by enzymatic methods using a Hitachi 7600-110 automated chemistry analyzer (Hitachi, Tokyo, Japan). Participants were requested to submit early morning samples of 5–20 mL urine. Ucot concentrations were measured by gas chromatographic mass spectrometry using a PerkinElmer Clarus 600T instrument (PerkinElmer, Waltham, MA, USA). The cut-off values of cotinine-verified current smoking ranged between 31.5 and 550 ng/mL in previous studies [19,20,21]. We classified participants with Ucot concentrations above or equal to 50 ng/mL as cotinine-verified current smoker according to the recommendation by the Society for Research on Nicotine and Tobacco [22], which have been validated in previous studies [23,24,25].

### 2.3. Statistical Analysis

All statistical analyses were performed using SAS software, version 9.4 (SAS Institute Inc., Cary, NC, USA), which incorporates sample weights and adjusts for the complex sample design of the survey. All statistical tests were two-sided and statistical significance was determined at *p* < 0.05. The survey sample weights were used to produce estimates that are representative of Korean adult men and women. To examine the dose-response relationship between cotinine-verified smoking and high leukocyte count, Ucot concentrations were categorized into five groups as follows: A non-smoker group (<50.00 ng/mL) and four quartile groups for smokers (Q1: 50.00–589.29 ng/mL, Q2: 722.57–1,254.73 ng/mL, Q3: 1,177.92–1,826.40 ng/mL, and Q4: ≥1,826.41 ng/mL) for men and (Q1: 50.00–162.79 ng/mL, Q2: 162.80–556.62 ng/mL, Q3: 556.63–1,073.13 ng/mL, and Q4: ≥1,073.14 ng/mL) for women. The characteristics of the study population were summarized using the weighted one-way analysis of variance (ANOVA) test for continuous variables and weighted chi-square test for categorical variables. The odds ratios (ORs) and 95% confidence intervals (CIs) for high leukocyte count (≥75th percentile in the current sample, ≥7350 cells/μL for men and 6630 cells/μL for women) were calculated across the five groups of Ucot concentrations using multivariate logistic regression analysis after adjusting for confounding variables.

## 3. Results

The characteristics of the study population according to Ucot concentrations are described in Table 1 and Table 2. The mean values of age and mean arterial pressure were highest in nonsmokers for both men and women. The prevalence of self-reported current smoking was 43.5% in men and 6.1% in women, whereas the prevalence of cotinine-verified current smoking was 46.5% in men and 11.5% in women. The mean values of BMI were lowest in the 4th Ucot quartile for both men and women. The mean values of triglycerides were highest in the 4th Ucot quartile for both men and women. The proportion of alcohol drinking ≥two times per week was highest whereas the education level of college or above was lowest in the 4th Ucot quartile for both men and women. The proportion of highest household income (Q4) was highest in nonsmokers for men and 1st Ucot quartile for women.

**Figure 1:**
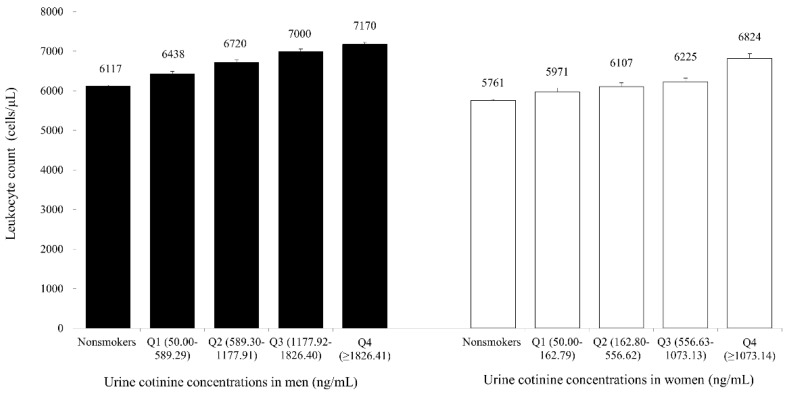
The mean values of leukocyte count according to urine cotinine concentrations (bars mean standard error).

Figure 1 shows the mean values of leukocyte counts according to the five groups of Ucot concentrations. The overall leukocyte count mean value gradually increased according to the grade of Ucot concentration: 6117, 6438, 6720, 7000, and 7170 cells/μL for men and 5761, 5971, 6107, and 6824 cells/μL for women (All *p* values < 0.001).

Table 3 shows the dose-response relationship between Ucot concentrations and high leukocyte counts. Compared with the cotinine-verified non-smoker group, the ORs (95% CIs) of high leukocyte count in terms of the quartiles of Ucot concentrations in current smokers were 1.70 (1.38–2.09), 2.35 (1.92–2.88), 3.54 (2.93–4.28), and 4.09 (3.39–4.94) for men and 1.02 (0.71–1.45), 1.42 (1.03–1.94), 1.58 (1.14–2.19), 3.91 (2.82–5.41) for women after adjusting for age, BMI, alcohol intake, education level, household income, occupation, and triglyceride level.

## 4. Discussion

In this large, population-based cross-sectional study, Ucot concentrations were independently and positively associated with systemic inflammation as measured by leukocyte counts in a dose-response manner for both men and women. Compared with the cotinine-verified non-smoker group, which was the reference group of this study, the odds ratio (OR) for high leukocyte count in the highest quartile group of Ucot concentrations was approximately four times higher after adjusting for potential confounding variables. The positive association between cotinine-verified smoking and leukocyte count is compatible with the findings of previous studies [26,27]. It has been demonstrated that current smokers have higher levels of inflammatory markers than people who have never smoked [28]. Kawada et al. reported that leukocyte count was higher in smokers and ex-smokers than in non-smokers, and elevated leukocyte counts were maintained for several years after smoking cessation [27]. A previous study also found a positive association between smoking status categorized into non-smoker, ex-smoker, and current smoker and leukocyte counts among 416,065 Korean adults [29]. However, most previous studies estimated smoke exposure using self-reported questionnaires, which have several limitations such as response inaccuracy or omissions of second-hand tobacco smoke exposure [12,13]. In the present study, we found the notable discrepancy between self-reported smoking and cotinine-verified smoking rate particularly in women. The prevalence of cotinine-verified smoking rate in women was approximately two times higher than that of self-reported smoking (11.5% vs. 6.1%), consistent with previous findings implicating that the smoking rate is likely to be underestimated on basis of self-reported questionnaire in studies targeting the female population [12,13]. As described earlier, the accurate analysis of dose-effect relationships of tobacco smoking could be affected by smoking habits such as the degree of inhalation, smoking cessation duration, and type of tobacco. To overcome these limitations, we used Ucot concentration, a reliable, quantitative biomarker of tobacco smoke exposure. Among the various biochemical markers of tobacco smoke exposure, Ucot has been commonly used in prevalent surveys to validate self-reported smoking status due to its higher stability and longer half-life [19].

Although the underlying biological mechanisms that explain smoking-induced increases in leukocyte count are not fully understood, there are several lines of evidence suggesting that tobacco smoking has the ability to evoke an inflammatory response through direct or indirect pathways. Tobacco smoking chronically stimulates the airway tract and subsequently may increase leukocyte count. Many toxins such as carbon monoxide, benzene, benzopyrene, and many other reactive oxidant substances during tobacco smoking activate inflammation in respiratory tracts in a direct manner, resulting in the production of potent inflammatory mediators such as tumor necrosis factor (TNF)-α, and interleukins [7,27,29,30]. Moreover, the pro-inflammatory cytokines induced by chronic exposure to tobacco smoke indirectly lead to systemic low-grade inflammation beyond the respiratory system [7,30,31,32].

In the present study, we used serum leukocyte count as an indicator of low-grade systemic inflammation. Leukocyte count is a simple, easy to obtain, and useful inflammatory marker in clinical practice. In addition to a marker of acute infection, high leukocyte count, even within the normal range, has been positively associated with all-cause mortality and incidence risk of cardiovascular diseases [4,5]. Moreover, numerous studies repeatedly demonstrated that leukocyte count is an independent predictor of the initiation and progression of inflammatory processes related to cardiometabolic diseases [2,3]. Thus, high leukocyte count could be used as a useful biomarker with reflecting low-grade systemic inflammation.

There are some limitations of the present study that should be considered when interpreting its findings. First, this study was a cross-sectional study by design and thus it was not possible to establish a causal relationship between Ucot concentration and leukocyte counts. However, it is more likely that smoking causes inflammation rather than inflammation causing smoking. Second, leukocyte counts in this study were only measured once, and thus it was not possible to determine whether an acute and brief episode of infection or chronic inflammation was responsible for the observed correlation. In order to reduce this potential error, we excluded subjects with leukocyte counts ≥10,000 cells/μL. Moreover, we did not exclude the presence of allergic diseases which may affect leukocyte count. Lastly, most of the tobacco users in Korean population are cigarette smokers, so our results may not be applied to other populations who mainly use other types of tobacco such as oral tobacco products. Despite these potential limitations, our study has also several strengths. First, the findings of the present study can be generalized because the KNHANES dataset represents the entire Korean population. Second, this study assessed objective tobacco smoking by Ucot concentrations and excluded the second-hand tobacco smoke exposure in non-smokers to minimize the information and misclassification bias on tobacco use. Lastly, this study controlled comprehensive confounding variables including both sociodemographic and laboratory parameters in regression analysis models, as compared to previous studies [26,28].

## 5. Conclusions

In conclusion, the present study confirmed a dose-response toxic effect of tobacco smoking, as measured by Ucot concentrations on low-grade systemic inflammation. Our results indicate that high leukocyte count could be another useful indicator of tobacco smoke exposure in a dose-response manner. Moreover, future prospective studies with a larger number of participants over a longer period of time are warranted to investigate the impact of smoking cessation on leukocyte count.

## Figures and Tables

**Table 1:** Characteristics of the study population according to urine cotinine concentrations in men.

	Total	Nonsmokers	Urine Cotinine Quartiles in Smokers (ng/mL)				
			Q1 (50.00–589.29)	Q2 (589.3–1177.91)	Q3 (1177.92–1826.40)	Q4 (≥1826.41)	*p*-Value
N	8655	4814	960	960	959	962	
Age (years)	42.2 (0.2)	43.9 (0.2)	43.3 (0.5)	40.0 (0.4)	40.9 (0.4)	41.3 (0.4)	<0.001
Body mass index (kg/m^2^)	24.2 (0.0)	24.3 (0.1)	24.3 (0.1)	24.2 (0.1)	24.2 (0.1)	23.7 (0.1)	0.004
Mean arterial pressure (mmHg)	92.4 (0.2)	92.7 (0.2)	91.9 (0.4)	91.2 (0.4)	92.3 (0.4)	92.1 (0.4)	0.014
Fasting plasma glucose (mg/dl)	98.8 (0.2)	99.3 (0.3)	99.5 (1.1)	99.5 (0.9)	98.3 (0.7)	95.9 (0.6)	<0.001
Triglyceride (mg/dl)	160.9 (1.7)	150.5 (2.4)	159.9 (4.9)	177.1 (5.0)	170.8 (6.1)	183.9 (5.3)	<0.001
HDL cholesterol (mg/dl)	47.5 (0.2)	47.7 (0.2)	48.7 (0.6)	47.2 (0.6)	46.6 (0.6)	46.4 (0.6)	0.075
Self-reported smoking status (%)							<0.001
Never smoker	23.8 (0.5)	42.9 (0.9)	5.0 (0.8)	0.9 (0.3)	0.2 (0.1)	0.5 (0.2)	
Ex-smoker	32.7 (0.5)	54.7 (0.8)	19.2 (1.5)	6.5 (1.1)	3.0 (0.6)	0.8 (0.2)	
Current smoker	43.5 (0.6)	2.4 (0.2)	75.8 (1.7)	92.6 (1.1)	96.8 (0.7)	98.7 (0.4)	
Alcohol intake (%)							<0.001
Non-drinker	7.8 (0.3)	10.2 (0.5)	4.3 (0.7)	4.2 (0.6)	6.3 (0.9)	5.6 (0.8)	
Once/week	54.1 (0.6)	58.5 (0.9)	52.3 (1.9)	54.1 (1.8)	45.5 (1.9)	44.6 (1.9)	
≥2 times/week	38.1 (0.6)	31.3 (0.8)	43.4 (1.9)	41.7 (1.8)	48.2 (1.9)	49.8 (1.9)	
Regular exercise (%)							0.532
None	48.8 (0.8)	48.5 (1.2)	47.1 (2.2)	49.2 (2.3)	49.2 (2.2)	50.7 (2.4)	
1–2 times/week	23.5 (0.7)	23.7 (0.9)	26.3 (1.9)	24.1 (1.8)	23.1 (1.8)	19.5 (1.8)	
≥3 times/week	27.7 (0.7)	27.8 (1.0)	26.6 (1.9)	26.7 (2.0)	27.7 (2.1)	29.8 (2.2)	
Education level (%)							<0.001
≤middle school	17.6 (0.5)	17.8 (0.6)	15.7 (1.2)	16.3 (1.4)	17.1 (1.3)	19.7 (1.4)	
High school	42.6 (0.7)	40.6 (0.9)	40.5 (2.0)	45.5 (1.9)	47.0 (1.9)	47.1 (1.9)	
≥college	39.8 (0.7)	41.6 (0.9)	43.8 (2.1)	38.2 (1.8)	35.9 (1.8)	33.2 (1.9)	
Household income (%)							0.027
1st quartile	10.9 (0.4)	10.4 (0.5)	11.6 (1.1)	12.5 (1.2)	11.5 (1.1)	10.4 (1.1)	
2nd quartile	24.8 (0.6)	24.1 (0.8)	24.1 (1.5)	27.2 (1.7)	25.0 (1.6)	25.6 (1.6)	
3rd quartile	31.3 (0.6)	30.3 (0.8)	32.4 (1.9)	32.0 (1.8)	31.0 (1.7)	34.8 (1.8)	
4th quartile	33.0 (0.8)	35.2 (1.0)	31.9 (1.9)	28.3 (1.7)	32.5 (1.8)	29.2 (1.7)	
Occupation (%)							<0.001
Unemployed	21.0 (0.5)	24.0 (0.8)	20.6 (1.6)	19.8 (1.5)	15.9 (1.4)	13.5 (1.3)	
Blue-collar workers	34,.8 (0.7)	31.7 (0.9)	30.9 (1.7)	35.0 (1.8)	41.7 (1.9)	46.0 (1.9)	
White-collar workers	44.2 (0.7)	44.3 (0.9)	48.5 (1.9)	45.2 (1.9)	42.4 (1.9)	40.5 (1.9)	

Data are expressed as the mean (SE) or percentage (SE). *p* values were calculated using weighted one-way ANOVA test or weighted chi-square test.

**Table 2:** Characteristics of the study population according to urine cotinine concentrations in women.

	Total	Nonsmokers	Urine Cotinine Quartiles in Smokers (ng/mL)				
			Q1 (50.00–162.79)	Q2 (162.80–556.62)	Q3 (556.63–1073.13)	Q4 (≥1073.14)	*p*-value
N	10,342	9,329	275	276	276	276	
Age (years)	44.6 (0.2)	45.1 (0.2)	41.0 (1.0)	38.4 (1.0)	40.1 (0.9)	42.1 (0.9)	<0.001
Body mass index (kg/m^2^)	23.1 (0.0)	23.1 (0.0)	23.1 (0.3)	22.7 (0.2)	22.9 (0.2)	22.2 (0.2)	0.004
Mean arterial pressure (mmHg)	86.3 (0.2)	86.5 (0.1)	84.8 (0.7)	84.9 (0.8)	85.1 (0.7)	84.8 (0.7)	0.004
Fasting plasma glucose (mg/dl)	95.1 (0.2)	95.3 (0.2)	94.4 (1.1)	93.8 (1.0)	94.6 (1.2)	93.4 (1.1)	0.313
Triglyceride (mg/dl)	108.6 (0.9)	107.3 (0.8)	106.9 (5.9)	116.7 (5.9)	121.5 (5.2)	129.1 (7.0)	<0.001
HDL cholesterol (mg/dl)	54.7 (0.2)	54.6 (0.2)	55.2 (1.9)	56.4 (1.3)	55.9 (1.4)	55.1 (1.2)	0.519
Self-reported current smoking (%)							<0.001
Never smoker	87.5 (0.4)	93.7 (0.3)	74.2 (3.4)	36.5 (3.4)	27.0 (3.1)	26.3 (3.3)	
Ex-smoker	6.4 (0.3)	5.9 (0.3)	11.5 (2.3)	13.8 (2.6)	7.1 (1.8)	7.8 (1.8)	
Current smoker	6.1 (0.3)	0.4 (0.1)	14.4 (2.9)	49.7 (3.6)	65.9 (3.4)	65.9 (3.4)	
Alcohol intake (%)							<0.001
Non-drinker	18.4 (0.5)	19.4 (0.5)	11.5 (2.2)	9.2 (1.7)	18.0 (2.6)	8.1 (1.9)	
Once/week	68.8 (0.6)	70.0 (0.6)	65.8 (3.6)	59.3 (3.3)	57.8 (3.6)	58.4 (3.6)	
≥2 times/week	12.8 (0.4)	10.5 (0.4)	22.7 (3.3)	31.5 (3.1)	24.2 (3.1)	33.5 (3.5)	
Regular exercise (%)							0.758
None	59.7 0.9)	59.7 (0.9)	58.9 (3.6)	59.3 (4.2)	55.3 (4.2)	63.2 (4.3)	
1–2 times/week	15.8 (0.6)	15.9 (0.6)	18.7 (2.8)	14.1 (3.0)	14.3 (3.2)	14.6 (3.4)	
≥3 times/week	24.5 (0.8)	24.4 (0.8)	22.4 (3.0)	26.6 (3.7)	30.4 (3.7)	22.2 (3.6)	
Education level (%)							<0.001
≤middle school	29.4 (0.6)	30.1 (0.6)	23.9 (2.9)	18.0 (2.9)	27.3 (2.8)	29.8 (3.0)	
High school	38.4 (0.7)	33.7 (0.6)	42.8 (3.5)	53.5 (3.6)	53.8 (3.5)	52.1 (3.4)	
≥college	32.2 (0.7)	33.2 (0.7)	33.3 (3.5)	28.5 (3.3)	18.9 (2.7)	18.0 (2.7)	
Household income (%)							<0.001
1st quartile	14.9 (0.4)	14.8 (0.4)	13.8 (2.3)	13.8 (2.3)	16.6 (2.4)	19.8 (2.6)	
2nd quartile	26.0 (0.6)	25.1 (0.6)	30.9 (3.3)	30.5 (3.3)	34.3 (3.5)	36.1 (3.4)	
3rd quartile	28.8 (0.6)	29.4 (0.6)	23.2 (2.8)	24.8 (3.2)	21.0 (2.7)	24.4 (3.2)	
4th quartile	30.3 (0.8)	30.6 (0.8)	32.1 (3.4)	30.9 (3.3)	28.1 (3.7)	19.7 (2.9)	
Occupation (%)							0.714
Unemployed	45.9 (0.6)	48.2 (0.6)	46.2 (3.6)	46.7 (3.6)	45.7 (3.6)	43.3 (3.5)	
Blue-collar workers	15.7 (0.5)	15,7 (0.5)	15.3 (2.3)	12.3 (2.3)	15.7 (3.3)	17.8 (2.6)	
White-collar workers	35.4 (0.6)	36.1 (0.6)	38.5 (3.7)	41.0 (3.5)	38.6 (3.4)	38.9 (3.5)	

Data are expressed as the mean (SE) or percentage (SE). *p* values were calculated using weighted one-way ANOVA test or weighted chi-square test.

**Table 3:** Odds ratios and 95% confidence intervals for high leukocyte count according to urine cotinine concentrations.

		Urine Cotinine Quartiles in Smokers (ng/mL)				
Men	Nonsmokers	Q1	Q2	Q3	Q4	*p* for trend
Model 1	1.00	1.56 (1.38–1.90)	2.29 (1.89–2.77)	3.37 (2.83–4.02)	3.85 (3.23–4.57)	<0.001
Model 2	1.00	1.64 (1.34–2.01)	2.37 (1.95–2.89)	3.42 (2.85–4.11)	3.80 (3.17–4.55)	<0.001
Model 3	1.00	1.70 (1.38–2.09)	2.35 (1.92–2.88)	3.54 (2.93–4.28)	4.09 (3.39–4.94)	<0.001
Women	Nonsmokers	Q1	Q2	Q3	Q4	*p* for trend
Model 1	1.00	1.02 (0.72–1.42)	1.36 (1.02–1.81)	1.57 (1.16–2.11)	4.17 (3.11–5.60)	<0.001
Model 2	1.00	0.99 (0.70–1.41)	1.46 (1.07–1.98)	1.59 (1.16–2.17)	4.32 (3.14–5.96)	<0.001
Model 3	1.00	1.02 (0.71–1.45)	1.42 (1.03–1.94)	1.58 (1.14–2.19)	3.91 (2.82–5.41)	<0.001

Model 1: Adjusted for age and body mass index. Model 2: Adjusted for age, body mass index, and alcohol intake. Model 3: Adjusted for age, body mass index, alcohol intake, education level, household income, occupation and triglyceride level.
